# Quality in clinical research: an observational study of randomisation techniques in urological and general surgical studies

**DOI:** 10.1097/JS9.0000000000001859

**Published:** 2024-06-27

**Authors:** Nicholas T.J. Raison, Simone Giona, Oliver Brunckhorst, Alexander Cohen, Gordon Muir

**Affiliations:** a Department of Urology, King’s College Hospital NHS Foundation Trust; b School of Biomedical Engineering and Imaging Sciences, King’s College London; c Department of Haematological Medicine, Guy’s and St Thomas’ Hospitals NHS Foundation Trust; d MRC Centre for Transplantation, Guy’s Hospital Campus, King’s College London, London; e Department of Urology, Frimley Park Hospital, Camberley, UK

Highlights

- This study objectively evaluated the quality of randomised controlled trials by assessing randomisation outcomes.
- Reported numbers of participants randomised to each arm in 148 studies were compared to expected numbers if simple randomisation was performed.
- Analysis showed that it is very unlikely (*P*<0.0001) that randomisation occurred as reported in all studies.

The randomised controlled trial (RCT) has long been considered the non-plus ultra of empirical research, relying on an equal distribution of confounding factors between arms. The Serious risks of bias and error from weak or flawed trial methodologies are now widely recognised^1^. The Consolidated Statement of Reporting Trials (CONSORT) statement provides a checklist of items that should be reported by RCTs. Reviews have systematically evaluated various bodies of literature against these established standards with persistently poor results.^2,3^. It is evident that deficiencies persist in the understanding and reporting of trial methodologies, particularly regarding randomisation and allocation procedures^4^.

Even these critical reviews are constrained by their reliance on authors’ accurate reporting of their trial methodologies. The primary objective of this study was to provide an objective assessment of the quality and reliability of RCT methodologies, specifically focusing on the outcomes of allocation following simple randomisation. Whilst straightforward to execute, a major limitation of simple randomisation is unequal allocation, especially in small studies, which can complicate trial outcomes. Yet despite the likelihood of imbalanced allocation, studies reporting equal group sizes remain prevalent. By comparing the expected and observed numbers of simply randomized studies with equal group sizes, the study aimed to quantify the likelihood that reported results accurately reflect actual allocation processes.

We reviewed all poster presentations of surgical RCTs presented at nine international surgical conferences covering urology and general surgery between 2012 and 2017 (see Supplementary Information, Supplemental Digital Content 1, http://links.lww.com/JS9/C896). Two authors independently identified studies before each conference. Studies were included if they labelled themselves as RCTs in the title or abstract or reported the random allocation of patients to study arms. Studies were excluded if they did not report original data, details of randomisation or allocation were not available and trials did not involve human participants or ongoing trials. The study was conducted in accordance with STROBE reporting guidelines^5^.

Data were collected in a stepwise manner. Abstracts were reviewed for randomisation technique and the number of patients allocated to each arm. The CONSORT definition of simple randomisation was used. Where possible, during each conference, presentations were attended and study authors were directly questioned as to the randomisation technique and allocation outcomes. For those cases where authors were unavailable or did not attend, they were contacted by e-mail (initially the presenting author then all authors for whom contact details were available). If no e-mail address or contact details were provided with the poster, internet searches were undertaken to find contact information. If no e-mail address was found or there was no response, subsequent full publications were reviewed to identify the randomisation technique. The authors were then contacted to confirm the same study design was used in the study presented and the manuscript published. Studies for which randomisation details could not be confirmed by any such method were excluded.

For all included studies, the following data were collected: randomisation technique; number of study arms and number of participants allocated to each arm; multicentre or single centre trial design; presence of external funding; prospective trial registration and type of trial (medical/surgical/mixed). We defined ‘Medical’ trials as those assessing the use of a pharmacological agent or drug. ‘Surgical’ trials are those directly assessing a surgical intervention. Mixed trials were those that either combined a medical and surgical intervention, used a non-surgical intervention (e.g. urinary catheter), or involved imaging modalities.

Analysis of the randomisation outcomes was performed by comparing the expected number of balanced studies against the observed number of balanced studies. A study was judged to be balanced if the difference between the number of patients in each trial arm(s) was 1 or 0. Studies with unequal randomisation ratios (e.g. 2:1) were normalised to the largest group. The expected number of balanced studies and a one-sided *P*-value were calculated to test if simple randomisation was effective in each study. A total of 10 000 Monte Carlo simulations under the assumption of simple randomisation were run for each study. The expected number of balanced studies was calculated as the mean number of balanced studies per simulation cycle. One-sided *P* values were calculated as the relative proportion of simulation cycles in which the number of balanced studies was equal to or greater than the number of observed balanced studies. All simulations were performed using the Stata MP Version 14.2 (StataCorp, LP). Our primary analysis included all studies. Sensitivity analyses were performed with studies stratified by study design, single centre vs. multicentre; type and the presence of external sponsorship.

Across the nine conferences, 504 RCTs were presented, of which 345 met our inclusion criteria. The specific randomisation technique was identified in 241 studies. 148 studies used simple randomisation to allocate 26 510 patients. The randomisation technique could not be identified by any method in 104 studies despite focussed efforts (Fig. 1).

**Figure 1:**
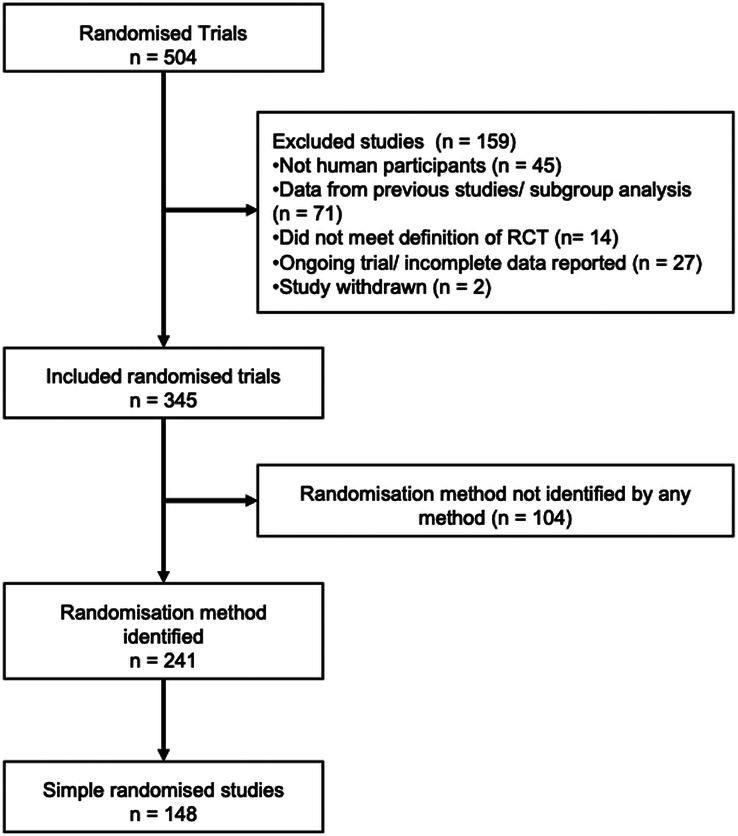
Study flow diagram.

Primary outcome analysis and sensitivity analyses are shown in Table 1. Analyses of the primary outcome and all subgroups demonstrate a probability of *P*<0.0001 that participant assignment was based on simple randomisation in all cases. The observed numbers of balanced studies greatly exceeded the expected numbers in all analyses.

**Table 1:** Results of primary analysis and sensitivity analyses.

Study type	Expected number of balanced studies (mean)	Observed number of balanced studies (*n*)	Total number of studies (*n*)	One-sided *P*
All studies	14	90	148	<0.0001
Number of centres				
Multicentre	2	10	25	<0.0001
Single centre	12	80	123	<0.0001
Industry sponsored?				
No	10	74	119	<0.0001
Yes	3	16	29	<0.0001
Type of intervention				
Medical	2	11	22	<0.0001
Surgical	7	50	76	<0.0001
Mixed	4	29	50	<0.0001

Our analysis of 148 RCTs randomising 26 510 patients has demonstrated that it is highly likely that a significant proportion of included studies did not perform randomisation as described. No difference was seen across study design, presence of funding, or study type. Almost one-third of studies were excluded as we could not find any information on randomisation despite various and repeated efforts to contact study authors. Numerous examples of a lack of understanding of trial methodology were noted during off-record interviews with authors. Authors did not know randomisation techniques, and many reported flawed techniques like patient preference or alternating between techniques, and two admitted that randomisation was not performed! That these results are restricted to poster presentations, possibly leading to higher proportions of low-quality studies, should be noted. However, all trials were peer-reviewed, and results were reported in international journals. Our results raise important questions on the quality and reliability of clinician-led medical research. While we cannot definitively determine the reasons behind these results, it is crucial to acknowledge and guard against the possibility of basic statistical flaws, especially in single-centre RCTs.

## Ethical approval

Ethical approval is not required.

## Consent

Consent is not required for this study.

## Source of funding

This study had no external funding.

## Author contribution

G.M. and N.T.J.R.: concept and design; N.T.J.R. and S.G.: acquisition of data; N.T.J.R., S.G., O.B., A.C. and G.M.: analysis of data; N.T.J.R., S.G. and O.B.: drafting of the manuscript; A.C. and G.M.: critical revision; G.M.: supervision.

## Conflicts of interest disclosure

The authors declare no conflicts of interest.

## Research registration unique identifying number (UIN)

Not applicable.

## Guarantor

Mr G. Muir.

## Data availability statement

Participants (authors of included studies) of this study did not give written consent for their data to be shared publicly, and all discussions were ‘off the record’, given the sensitive nature of the research. As a result, specific study data is not available. Cumulative data from all studies is available on request from study authors.

## Provenance and peer review

Not commissioned

## Supplementary Material

SUPPLEMENTARY MATERIAL
