# Supplementary material for: Quality in clinical research: an observational study of randomisation techniques in urological and general surgical studies
Source: Int J Surg. 2024 Jun 27;110(10):6843–5. doi: 10.1097/JS9.0000000000001859 (PMC11486936; doi:10.1097/JS9.0000000000001859)
Supplement: SUPPLEMENTARY MATERIAL [file js9-110-6843-s001.docx]

Supplementary Online Table 1: Surgical and Urological Conferences Included in this Study

American Urological Association Annual Congress 2012

American Urological Association Annual Congress 2013

American Urological Association Annual Congress 2014

American Urological Association Annual Congress 2015

American Urological Association Annual Congress 2016

European Association of Urology Annual Congress 2012

European Association of Urology Annual Congress 2013

European Association of Urology Annual Congress 2015

European Association of Urology Annual Congress 2016

World Congress of Surgery 2017
